# 2025 Updated version v1.0 SEOM-GEMCAD-TTD clinical guidelines for the systemic treatment of metastatic colorectal cancer (2022)

**DOI:** 10.1007/s12094-025-03860-x

**Published:** 2025-02-25

**Authors:** Ana Fernández Montes, Vicente Alonso, Enrique Aranda Aguilar, Elena Élez, Pilar García Alfonso, Cristina Grávalos Castro, Joan Maurel, Ruth Vera García, Rosario Vidal Tocino, Jorge Aparicio Urtasun

**Affiliations:** 1 Medical Oncology Department, Complexo Hospitalario Universitario, Ourense (CHUO), C/ Ramón Puga, 56, 32005 Ourense, Spain; 2 Medical Oncology Department, Hospital Universitario Miguel Servet, Saragossa, Spain; 3 Medical Oncology Department, Hospital Universitario Reina Sofía, Córdoba, Spain; 4 Medical Oncology Department, Hospital Universitario Vall D’Hebron, Barcelona, Spain; 5 Medical Oncology Department, Hospital General Universitario Gregorio Marañón, Madrid, Spain; 6 Medical Oncology Department, Hospital Universitario, 12 de Octubre, Madrid, Spain; 7 Medical Oncology Department, Hospital Clínic, Barcelona, Spain; 8 Medical Oncology Department, Hospital Universitario de Navarra, Pamplona, Spain; 9 Medical Oncology Department, Complejo Asistencial Universitario, Salamanca, Spain; 10 Medical Oncology Department, Hospital Universitari I Politècnic la Fe, Valencia, Spain

## Summary

- Incidence and epidemiology
- Methodology
- Diagnosis, pathology and molecular biology
- Staging
- Management of liver limited disease
- Management of metastatic disease

V1.0 26 December 2024.

## Incidence and epidemiology

- **Incidence and mortality**
  - Third most common cancer worldwide (2022: 1.93 million cases). First in Spain (2024: 44,294).
  - Second highest cancer mortality (2020: 935,173 deaths). Second in Spain (2022: 15,198).
- **Metastatic disease**
  -  ~ 20% of patients have metastases at diagnosis.
  - 50% of initially localized cases may develop metastases.
  - Non-curable in most cases; median survival under 20–30 months.
- **Sporadic vs. Familial CRC**
  - 75–80% cases are sporadic.
  - 20% have familial aggregation.
  - 5–7% linked to hereditary syndromes (e.g., Lynch syndrome).
  - Colorectal cancer is on the rise in individuals under 50 years old, representing a significant public health concern.
- **Risk factors**
  - **Primary:** Aging
  - **Others:** Inflammatory bowel disease, colonic polyps
  - **Modifiable factors:** high red/processed meat intake, low fiber diet, alcohol, tobacco, obesity and sedentary lifestyle.

V1.0 26 December 2024.

## Methodology

This guideline is based on a systematic review of relevant published studies and with the consensus of ten treatment expert oncologists from Spanish cooperative groups GEMCAD and TTD and SEOM (Spanish Society of Medical Oncology).

The Infectious Diseases Society of America-US Public Health Service Grading System for Ranking Recommendations in Clinical Guidelines has been used to assign levels of evidence and grades of recommendation.

**Table:** 

I	Evidence from **at least one large randomised, controlled trial** of good methodological quality (low potential for bias) or **meta-analyses** of well-conducted randomised trials without heterogeneity	A	S**trong evidence** for efficacy with a **substantial clinical benefit, strongly** recommended
II	**Small randomised trials or large randomised trials with a suspicion of bias** (lower methodological quality) or meta-analyses of such trials or of trials with demonstrated heterogeneity	B	**Strong or moderate** evidence for efficacy but with a **limited** clinical benefit, **generally recommended**
III	**Prospective** cohort studies	C	**Insufficient evidence** for efficacy or **benefit does not outweigh the risk** or the disadvantages (adverse events, cost, etc.), **optional**
IV	**Retrospective** cohort studies or **case–control** studies	D	**Moderate** evidence against efficacy or for adverse outcome, generally **not recommended**
V	Studies **without control group**, case reports, expert opinions	E	**Strong** evidence against efficacy or for adverse outcome, **never recommended**

*LoE* Level of evidence, *GoR* grade of recommendation.

Dykewicz CA. Clin Infect Dis 2001; 33: 139–144 (Adapted from: Gross PA et al. Clin Infect Dis 1994; 18: 421).

V1.0 26 December 2024.

## Diagnosis, pathology and molecular biology

- A complete colonoscopy with biopsy to confirm the diagnosis is mandatory. Virtual colonoscopy is an alternative to detect potential synchronous colorectal lesions if a full colonoscopy is not feasible [I, A].
- CT scan of the chest, abdomen, and pelvis is the best technique to assess distant metastases [IV, A].
- MRI and PET-CT may be considered in selected cases [IV, B].
- Patients with mCRC should be evaluated by a multidisciplinary team to define patient management: resectable, potentially resectable and unresectable disease [III, A].
- The recommended staging system is that of the eighth edition of the AJCC [I, A]
- Resection of an asymptomatic primary tumour in patients with unresectable metastatic disease is not recommended as standard of care [I, D].

V1.0 26 December 2024.

## Diagnosis, pathology and molecular biology

- RAS exons (KRAS/NRAS) 2, 3, and 4, and BRAF V600E mutations should be tested at the time of mCRC diagnosis [I, A].
- Assessment of mismatch repair deficiency (IHC or MSI) is recommended to assist genetic counseling for Lynch syndrome [II, B] and mandatory for its predictive value of benefit from ICI [I, A].
- Identification of HER 2 amplification or overexpression [III, C] and NTRK fusions are recommended in subsequent lines for access to clinical trials with targeted therapies and to detect those who may benefit from targeted therapy [III, A].
- Liquid biopsy might be considered to monitor emergent mutations of resistance to targeted therapy, especially prior to re-challenge with anti-epidermal growth factor receptor (anti-EGFR) treatment, though this is not supported yet by our national authorities [II, B].
- Testing for DPYD deficiency is strongly recommended prior to initiate fluoropyrimidine-based chemotherapy [III, A]. UGT1A1 is recommended prior irinotecan-based chemotherapy.
- When single or multigene tumour testing is available and applicable, testing for *KRAS* G12C [I, A], and *POLE* mutations [III, C] as well as for genomic aberrations for which targeted therapeutics are approved in tumour-agnostic indications [*NTRK* fusions, *RET* fusions, TMB-H] is advised [III, C].

V1.0 26 December 2024.

## Staging (TNM 8th edition)

### Primary tumor (T)

- **TX**: Primary tumor cannot be assessed.
- **T0**: No evidence of primary tumor.
- **Tis**: Carcinoma in situ; cancer confined to the mucosa without invasion of the submucosa.
- **T1**: Tumor invades the submucosa.
- **T2**: Tumor invades the muscularis propria.
- **T3**: Tumor invades through the muscularis propria into pericolorectal tissues without reaching other organs.
- **T4a**: Tumor perforates the visceral peritoneum.
- **T4b**: Tumor directly invades or adheres to other organs or structures.

### Regional lymph nodes (N)

- **NX**: Regional lymph nodes cannot be assessed.
- **N0**: No regional lymph node metastasis.
- **N1**: Metastasis in 1 to 3 regional lymph nodes:
  - **N1a**: Metastasis in 1 regional lymph node.
  - **N1b**: Metastasis in 2 to 3 regional lymph nodes.
  - **N1c**: Tumor deposits in the subserosa, mesentery, or non-nodal pericolorectal tissues without regional lymph node involvement.
- **N2**: Metastasis in 4 or more regional lymph nodes:
  - **N2a**: Metastasis in 4 to 6 regional lymph nodes.
  - **N2b**: Metastasis in 7 or more regional lymph nodes.

### Distant metastasis (M)

- **M0**: No distant metastasis.
- **M1**: Distant metastasis present:
  - **M1a**: Metastasis confined to one organ or site (e.g., liver, lung, ovary, or non-regional lymph nodes).
  - **M1b**: Metastasis in more than one organ/site or the peritoneum.
  - **M1c**: Peritoneal metastasis with or without other organ involvement.

V1.0 26 December 2024.

## Liver-limited CRC

### Resectable disease


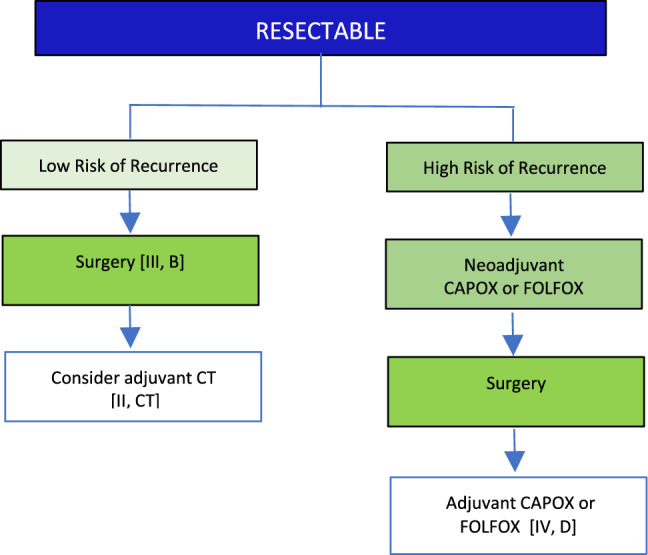


V1.0 26 December 2024.

## Liver-limited CRC

### Potentially resectable disease


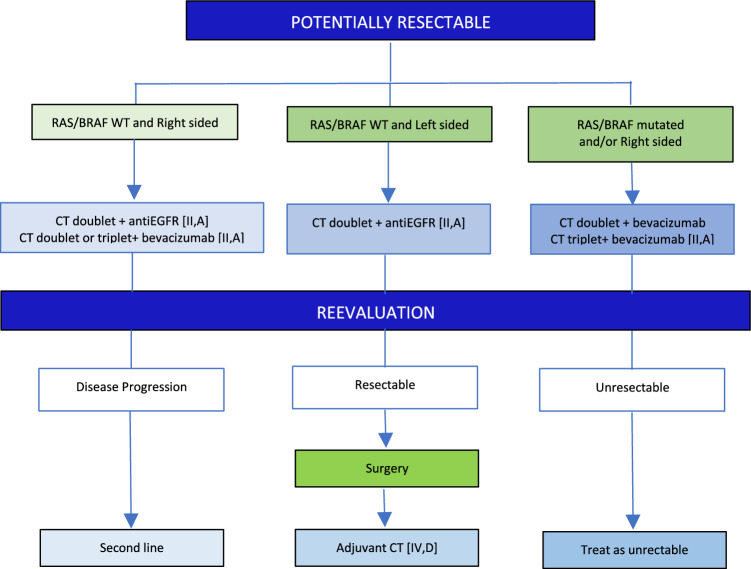


V1.0 26 December 2024.

## Metastatic disease: 1st line


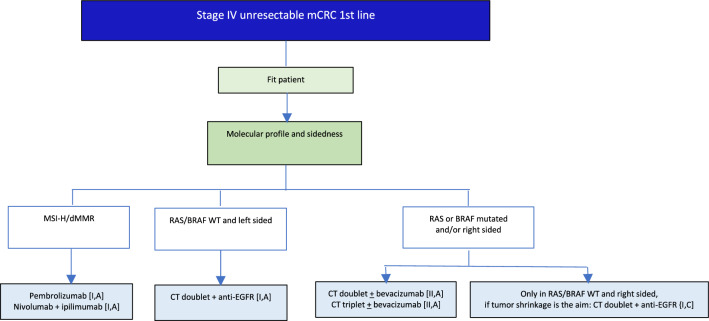


V1.0 26 December 2024.

## Metastatic disease: 1st line


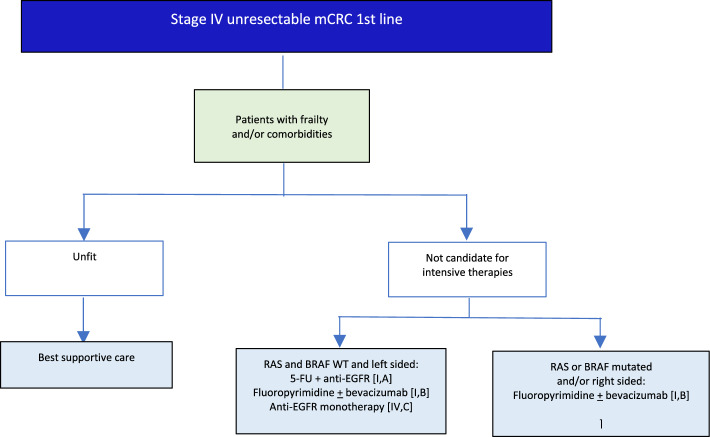


V1.0 26 December 2024.

## Metastatic Disease: 2nd line


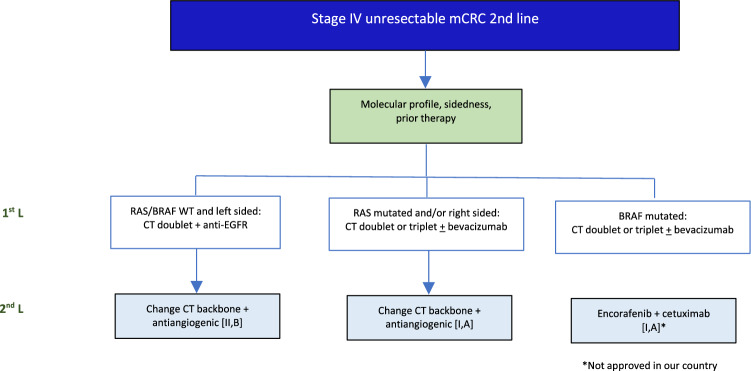


V1.0 26 December 2024.

## Metastatic disease: 3rd line and beyond


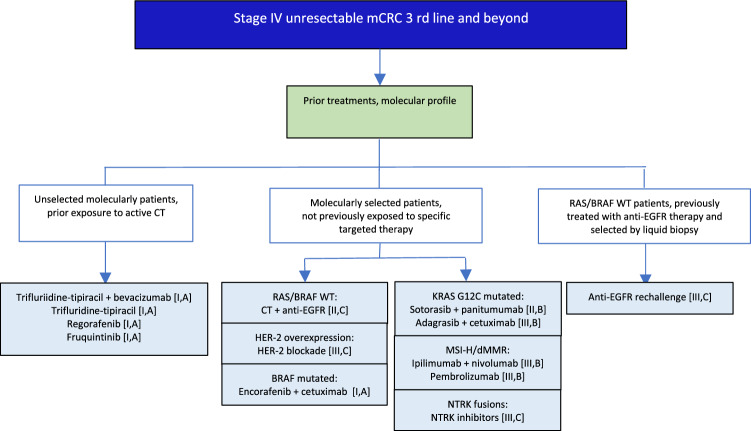


V1.0 26 December 2024.

## Follow-up

- For patients receiving active treatment, radiological evaluation should be carried out every 8–12 weeks, including (in most cases) CT scan or MRI, as well as the measurement of CEA levels [IV, B].
- Patients with a radically resected metastatic disease with potential for cure merit more intense monitoring initially with radiological assessment with CT (or MRI) and measurement of CEA levels every 3 months during the first 2 years and every 6 months thereafter [I, A].

V1.0 26 December 2024.

